# Development and Validation of a Multiplex Reverse Transcription PCR Assay for Simultaneous Detection of Three Papaya Viruses

**DOI:** 10.3390/v6103893

**Published:** 2014-10-21

**Authors:** Decai Tuo, Wentao Shen, Yong Yang, Pu Yan, Xiaoying Li, Peng Zhou

**Affiliations:** 1 Key Laboratory of Biology and Genetic Resources of Tropical Crops, Ministry of Agriculture, Institute of Tropical Bioscience and Biotechnology Chinese Academy of Tropical Agricultural Sciences, Haikou 571101, China; E-Mails: 273986569@qq.com (D.T.); 340014852@qq.com (Y.Y.); yanpu@itbb.org.cn (P.Y.); lixiaoying@itbb.org.cn (X.L.); 2 College of Agriculture, Hainan University, Haikou 570228, China

**Keywords:** PRSV, PLDMV, PapMV, multiplex RT-PCR, detection, papaya

## Abstract

*Papaya ringspot virus* (PRSV), *Papaya leaf distortion mosaic virus* (PLDMV), and *Papaya mosaic virus* (PapMV) produce similar symptoms in papaya. Each threatens commercial production of papaya on Hainan Island, China. In this study, a multiplex reverse transcription PCR assay was developed to detect simultaneously these three viruses by screening combinations of mixed primer pairs and optimizing the multiplex RT-PCR reaction conditions. A mixture of three specific primer pairs was used to amplify three distinct fragments of 613 bp from the *P3* gene of PRSV, 355 bp from the *CP* gene of PLDMV, and 205 bp from the *CP* gene of PapMV, demonstrating the assay’s specificity. The sensitivity of the multiplex RT-PCR was evaluated by showing plasmids containing each of the viral target genes with 1.44 × 10^3^, 1.79 × 10^3^, and 1.91 × 10^2^ copies for the three viruses could be detected successfully. The multiplex RT-PCR was applied successfully for detection of three viruses from 341 field samples collected from 18 counties of Hainan Island, China. Rates of single infections were 186/341 (54.5%), 93/341 (27.3%), and 3/341 (0.9%), for PRSV, PLDMV, and PapMV, respectively; 59/341 (17.3%) of the samples were co-infected with PRSV and PLDMV, which is the first time being reported in Hainan Island. This multiplex RT-PCR assay is a simple, rapid, sensitive, and cost-effective method for detecting multiple viruses in papaya and can be used for routine molecular diagnosis and epidemiological studies in papaya.

## 1. Introduction

Papaya (*Carica papaya* L.) is an important fruit crop grown widely in tropical and subtropical regions [1]. However, several viruses pose a serious threat to papaya production. These include *potyviruses*, such as *Papaya ringspot virus* (PRSV) [2], *Papaya leaf distortion mosaic virus* (PLDMV) [3], and *Zucchini yellow mosaic virus* (ZYMV) [4]; the *potexvirus*, *Papaya mosaic virus* (PapMV) [5]; the *geminiviruses*, *Papaya leaf curl virus* (PaLCV) [6], *Papaya leaf crumple virus* (PaLCrV) [7], and *Croton yellow vein mosaic virus* (CYVMV) [8]; the *rhabdoviruses*, *Papaya droopy necrosis virus* (PDNV) [9] and *Papaya apical necrosis virus* (PANV) [10]; the *tospovirus*, *Tomato spotted wilt virus* (TSWV) [11]; and the *sobemovirus*, *Papaya lethal yellowing virus* (PLYV) [12,13]. Among the known papaya viruses, PRSV, PLDMV, and PapMV have been detected on Hainan Island, China [5,14,15]. Currently, PRSV is considered the most widespread and destructive disease damaging papaya production in China [15]. However, the presence of PLDMV was confirmed recently in commercialized PRSV-resistant transgenic papaya on Hainan and Taiwan [14,16], indicating a potential threat to papaya in China and abroad. In contrast, PapMV has been considered of minor importance because it is rarely found in the field [17,18]. These three viruses cause similar symptoms in papaya, such as mosaic, yellow-green leaf discoloration and distortion on leaves, water-soaking streaks on petioles, and ring-spots on fruits, making it difficult to distinguish among PLDMV, PRSV, and PapMV without further testing. Furthermore, mixed infections of PRSV and PLDMV or PapMV have been reported in papaya in Taiwan and the Philippines, respectively [16,17]. Therefore, to make rapid diagnoses and limit disease spreading, it is necessary to develop an effective and rapid detection method for these viral infections in papaya.

Currently, several diagnostic methods have been developed for the detection of PRSV, PLDMV, or PapMV, such as enzyme-linked immunosorbent assay (ELISA), Western blotting, reverse transcription-polymerase chain reaction (RT-PCR), real time RT-PCR, and reverse transcription loop-mediated isothermal amplification (RT-LAMP) [17,18,19,20,21,22,23,24,25,26]. However, these techniques only can detected individual papaya viruses in each reaction and are time-consuming, laborious, and costly, especially when large numbers of samples need to be tested for mixed infections. These limitations can be overcome by employing a multiplex reverse transcription polymerase chain reaction method (multiplex RT-PCR), which can amplify simultaneously more than one target sequences of different viruses by several primer pairs in one signal reaction, offering a significant time and cost-saving advantage [27,28]. To date, multiplex RT-PCR has been used widely to diagnose infection in garlic, cassava, pear, cherry, apple, and potato plants by related plant viruses [21,22,23,29,30,31,32,33,34,35]. However, this method has not been applied to the simultaneous detection of PRSV, PLDMV, and PapMV. In this study, the development and evaluation of a multiplex RT-PCR assay for the simultaneous detection and differentiation of PRSV, PLDMV, and PapMV from papaya is described.

## 2. Materials and Methods

### 2.1. Viruses

PRSV (Accession No. HQ424465), PLDMV (Accession No. JX974555), and PapMV (Accession No. JX524226) strains isolated from Hainan were used as the reference strains in this study [5,14,15]. These three virus isolates propagated persistently in papaya were used as positive controls for the optimization of all RT-PCR assays.

### 2.2. Primer Design

The full-length genomic sequences of PRSV including PRSV-P and PRSV-W isolates were obtained from GenBank [36] (Accession No. AB369277, AY010722, AY027810, AY162218, AY231130, DQ340769, DQ340770, DQ340771, DQ374152, DQ374153, EF017707, EF183499, EU126128, EU475877, EU882728, HQ424465, JX448369, JX448370, JX448371, JX448372, JX448373, KC345609, KF791028, KF734962, X67673, and X97251) and aligned using Vector NTI Advance 11.0 software [37]. A highly conserved region (2774–4207 nt) covering the *HC-Pro* and *P3* genes was chosen as a suitable target to design three primers specific for PRSV (Table 1). 

**Table 1:** List of primers used in the multiplex detection for PRSV, PLDMV, and PapMV.

Virus	Primer ^a^	Sequence (5’-3’)	Size (bp)	Target Gene	Location (nt) ^b^
PRSV	PRSV953-F	GCGATGCTCATAAACATACCTGA	953	*HC-Pro/P3*	2774–2796
	PRSV953-R	TGTACACAGTACTTCGGTGAGAAGTCGTA			3698–3726
	PRSV613-F	TTGTGTACGACTTCTCACCGAA	613	*P3*	3693–3714
	PRSV613-R	CGAATGTCATCCAAAGACTGATGATAAAC			4277–4305
	PRSV515-F	TTGTGTACGACTTCTCACCGAA	515	*P3*	3693–3714
	PRSV515-R	CACATCAATCATCATCAAAATTAATGT			4181–4207
PapMV	PapMV476-F	ATGGTAGCTGCTAAGGTTCCAGC	476	*CP*	6024–6046
	PapMV476-R	GACCCAGAAATTTGGCCTTTGGTGATG			6473–6499
	PapMV205-F	CCAAATTTGCCGCGTTCGACT	205	*CP*	6295–6315
	PapMV205-R	GACCCAGAAATTTGGCCTTTGGTGATG			6473–6499
PLDMV	PLDMV355-F	GGCATGTGGTTTATGATGCAAGGG	355	*CP*	9528–9551
	PLDMV355-R	GCTCCGTGTTCTCAGTCGCATT			9861–9882

^a^ F, sense primer; R, antisense primer. ^b^ The targeting nucleotide locations according to the complete genome sequence of PRSV (HQ424465), PapMV (JX524226), and PLDMV (JX974555).

Similarly, based on the alignment of the CP gene sequences of the PLDMV isolates (Accession No. JX974555, JX416282, AB088221, EU240889, EU240890, EU240888, EF675245, EU233272, AB092816, AB092815, and AB092814) or PapMV isolates (Accession No. AY017186, AY017187, AY017188, D00240, D13957, EF183500, and JX524226), with conserved regions identified using Vector NTI Advance 11.0 software [37], two primers specific for PapMV and one specific for PLDMV were designed (Table 1). In this study, these primers were designed to have similar annealing temperatures (50–58 °C) by Primer3 [38,39]. The specificity of each primer was evaluated using Primer-BLAST [40].

### 2.3. Nucleic Acid Extraction

The papaya leaves (50–100 mg) were homogenized with liquid nitrogen. Total RNA was extracted using TRIzol reagent (Life Technologies, Carlsbad, CA, USA) according to manufacturer’s protocol. The yield and the quality of RNA were analyzed using a NanoVue^TM^ Plus Spectrophotometer (GE Healthcare, Freiburg, Germany).

### 2.4. Reverse Transcription

The revere transcription (RT) reaction was carried out using TaKaRa RNA PCR Kit (AMV) Ver. 3.0 (Takara, Dalian, China) according to the manufacturer’s protocol. The reaction was performed in a 10 μL PCR master mixture consisting of 2 μL MgCl_2 _(25 mM), 1 μL 10 × RT Buffer (100 mM Tris-HCl (pH 8.3), 500 mM KCl), 3.25 μL RNase Free dH_2_O, 1 μL dNTP Mixture (each 10 mM), 0.25 μL RNase Inhibitor (40 U/μL), 0.5 μL AMV Reverse Transcriptase XL (5 μL), 0.5 μL Random 9 mers (50 pmol/μL), 0.5 μL Oligo dT-Adaptor Primer (2.5 pmol/μL) and 1 μL plant total RNA (~500 ng). The RT reaction was incubated for 10 min at 30 °C followed by 30 min at 42 °C, and then heated for 5 min at 95°C. The products were chilled on ice and stored at −20 °C for uniplex RT-PCR and multiplex PCR reactions.

### 2.5. The Uniplex PCR

Uniplex PCR for PRSV, PLDMV, or PapMV was carried out in a 25 μL mixture containing 12.5 μL 2 × *Eco Taq* PCR SuperMix (+dye) (TransGen Biotech, Beijing, China), 0.5 μL of specific primers (20 μM) (Table 1), 2 μL cDNA template, and 10.5 or 9.5 μL sterile deionized H_2_O. In a negative control reaction, nuclease free water or cDNA from healthy (virus-free) plant was used as the template. To determine the best annealing temperature for a correct amplification preventing non-specific products, a gradient PCR was performed in a TGradient Thermocycler (Biometra, Goettingen, Germany) using the following parameters: one cycle at 94 °C for 3 min; 35 cycles of denaturation at 94 °C for 30 s, annealing at 50–65 °C (with an increase of 2 °C per tested sample) for 30 s, and extension at 72 °C for 1 min; and a final extension at 72 °C for 5 min. PCR products were examined by electrophoresing 10 μL aliquots on 1.5% agarose gels in TAE buffer. Each amplified viral target fragments was cloned into the pMD18-T vector (Takara), and the resulting plasmid construct pPRSV-953, pPRSV-613, pPRSV-515, pPLDMV-355, pPapMV-476, and pPapMV-205, was confirmed by sequencing (Life Technologies, Guangzhou, China) and BLASTN at NCBI [41].

### 2.6. Optimization of the Multiplex PCR Conditions

To simultaneously amplify all three viral targets in one reaction using three sets of virus-specific primers, six primer combinations (PRSV953-F/R + PapMV476-F/R + PLDMV355-F/R, PRSV613-F/R + PapMV476-F/R + PLDMV355-F/R, PRSV515-F/R + PapMV476-F/R + PLDMV355-F/R, PRSV953-F/R + PapMV205-F/R + PLDMV355-F/R, PRSV613-F/R + PapMV205-F/R + PLDMV355-F/R, PRSV515-F/R + PapMV205-F/R + PLDMV355-F/R), ten primer combinations for concentration (0.4 μM, 0.2 μM, or 0.1 μM of each primer pair), and multiplex PCR cycling parameters were optimized. For multiplex RT-PCR, artificial mixture of total plant RNA was prepared from the different papaya samples known to be infected with PRSV, PapMV or PLDMV. The multiplex PCR was performed using a PCR reaction mixture containing 12.5 μL 2 × *Eco Taq* PCR SuperMix (+dye) (TransGen Biotech), 0.4 μM, 0.2 μM, or 0.1 μM of each primers, 2 μL cDNA template and sterile deionized H_2_O was added to a final volume of 25 μL. The PCR program was an initial denaturation at 94 °C for 3 min; 35 cycles of at 94 °C for 30 s, annealing at 50–65 °C (with an increase of 2 °C per tested sample) for 30 s, and extension at 72 °C for 1 min; and a final extension at 72 °C for 5 min. The amplicons of 10 μL aliquots were determined by electrophoresis through 1.5% agarose-TAE gel.

### 2.7. The Sensitivity of the Uniplex PCR and Multiplex PCR Assays

To compare the sensitivity of multiplex RT-PCR *versus* uniplex RT-PCR, 10-fold serial dilutions of the purified plasmid pPRSV-613, pPLDMV-355, and pPapMV-205 were used simultaneously as templates in uniplex PCR and multiplex PCR reactions. The following formula was used to calculate the number of gene copies per μL in each dilution: copies/μL = (6.02 × 10^23^) × (Plasmid concentration (ng/μL) × 10^−9^)/(DNA length (bp) × 660) [42].

### 2.8. The Detection of Viruses in Field Samples by Uniplex RT-PCR and Multiplex RT-PCR

A total of 341 field papaya leaf samples showing symptoms such as mosaic, ring spots and distortion on leaves, and water soaking streaks on petioles, were collected from different geographical regions of Hainan Island (Table 2) between August 2013 and March 2014. Subsequently, these samples were simultaneously used to test PRSV, PLDMV, and PapMV by uniplex RT-PCR and multiplex RT-PCR.

## 3. Results

### 3.1. Specificity and Compatibility of Primer Pairs

**Figure 1:**
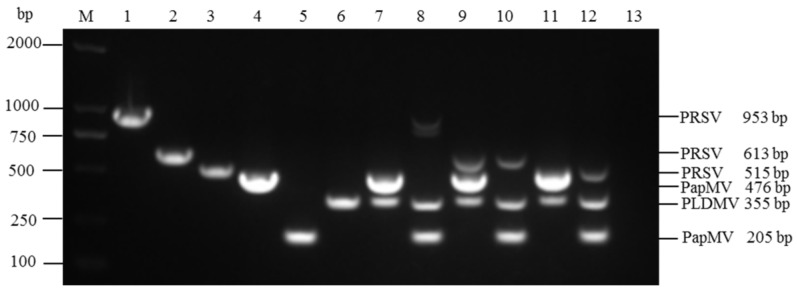
Determination of specificity and compatibility of six primer pairs used for the uniplex and multiplex PCR using different primer combinations to assay for PRSV, PLDMV, and PapMV. The amplified products were sizes of 953 bp, 613 bp, and 515 bp for PRSV; 476 bp and 205 bp for PapMV; and 355 bp for PLDMV. Lanes 1-6 indicate the uniplex PCR using primer pairs of PRSV953-F/R, PRSV613-F/R, PRSV515-F/R, PapMV476-F/R, PapMV205-F/R, and PLDMV355-F/R. Lanes 7–12 indicate the multiplex PCR using different primer combinations of three primer pairs specific for PRSV, PapMV, and PLDMV: (7) PRSV953-F/R, PapMV476-F/R, and PLDMV355-F/R; (8) PRSV953-F/R, PapMV205-F/R, and PLDMV355-F/R; (9) PRSV613-F/R, PapMV476-F/R, and PLDMV355-F/R; (10) PRSV613-F/R, PapMV205-F/R, and PLDMV355-F/R; (11) PRSV515-F/R, PapMV476-F/R, and PLDMV355-F/R; (12) PRSV515-F/R, PapMV205-F/R, and PLDMV355-F/R. Lane 13, negative control. Lane M, 2000 bp DNA marker.

In uniplex RT-PCR, the expected sizes of viral target genes for PRSV, PapMV, and PLDMV were amplified specifically for all of six primers (Table 1) designed in this study (Figure 1, Lane 1–6).

The sequences of amplified products were identical to the GenBank sequences (Accession No. HQ424465, JX524226, JX974555), respectively. For multiplex RT-PCR, the primer combination PRSV613-F/R + PLDMV355-F/R + PapMV205-F/R gave more clear and specific bands of target products compared to the rest combinations, and then the primer combination was selected for further optimization in multiplex RT-PCR (Figure 1, Lane 10).

### 3.2. Optimization of Multiplex RT-PCR

**Figure 2:**
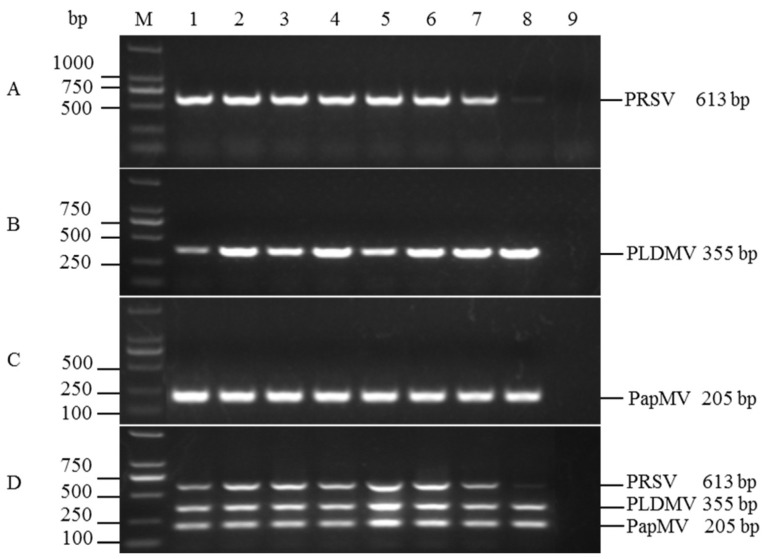
Optimization of the annealing temperature for uniplex RT-PCR of (**A**) PRSV (613 bp); (**B**) PLDMV (355 bp); and (**C**) PapMV (205 bp) and (**D**) multiplex RT-PCR assay. (**A**)–(**C**), uniplex RT-PCR; (**D**), multiplex RT-PCR. Lane M: DL2000 DNA Marker; 1: 50 °C; 2: 51.7 °C; 3: 53.6 °C; 4: 55.8 °C; 5: 57.9 °C; 6: 60.1 °C; 7: 62.2 °C; 8: 64.3 °C; 9: negative control (ddH_2_O).

**Figure 3:**
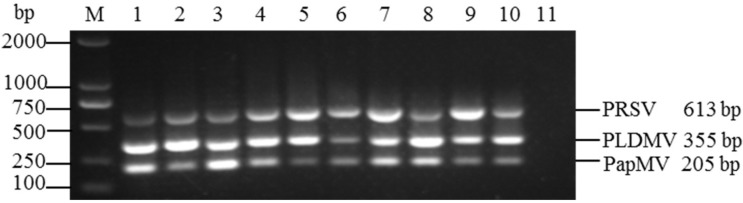
Optimization of the concentrations of three primer pairs used for the multiplex RT-PCR assay of PRSV, PLDMV, and PapMV. The amplified products of PRSV, PLDMV, and PapMV are indicated by the sizes of 613 bp, 355 bp, and 205 bp, respectively. Lanes 1–10 indicate the concentration combinations of three primers specific for PRSV, PLDMV, and PapMV: (1) 0.4:0.4:0.4 μM; (2) 0.4:0.4:0.2μM; (3) 0.4:0.2:0.4 μM; (4) 0.4:0.2:0.2 μM; (5) 0.4:0.2:0.1 μM; (6) 0.4:0.1:0.1 μM; (7) 0.4:0.1:0.2 μM; (8) 0.2:0.2:0.2 μM; (9) 0.2:0.1:0.1 μM; (10) 0.1:0.1:0.1 μM. Lane 11: negative control (ddH2O); Lane M: DL2000 DNA Marker.

A gradient RT-PCR was then performed to determine the optimal annealing temperature for uniplex RT-PCR and multiplex RT-PCR. In uniplex PCR assays, all primers performed well in the tested temperature range (50–64.3 °C) except at 64.3 °C for PRSV amplification (lane 8 in Figure 2A–C). Similarly, a faint amplification for PRSV was detected at 64.3 °C in multiplex RT-PCR (lane 8 in Figure 2D). According to amplification efficiency and specificity for the targeted viruses as detected by agarose gel electrophoresis, the optimal annealing temperature was determined to be 57.9 °C. The optimized cycle protocol of the RT-PCR was: 3 min at 94 °C; 35 cycles of 30 s at 94 °C, 30 s at 58 °C, and 1 min at 72 °C; and a final extension at 72 °C for 5 min. For the combinations of different concentrations of three primers, the amplification efficiencies of PRSV and PapMV varied at different combinations of primer concentrations, while that of PLDMV remained consistent and reliable (Figure 3). A balanced amplification, with similar fluorescence intensity for the bands corresponding to the three expected templates, was achieved when the primer concentrations were 0.4 μM for PRSV, 0.1 μM for PLDMV, and 0.2 μM for PapMV in the multiplex RT-PCR (Figure 3, Lane 7).

### 3.3. Sensitivities of the Uniplex and Multiplex RT-PCR

**Figure 4:**
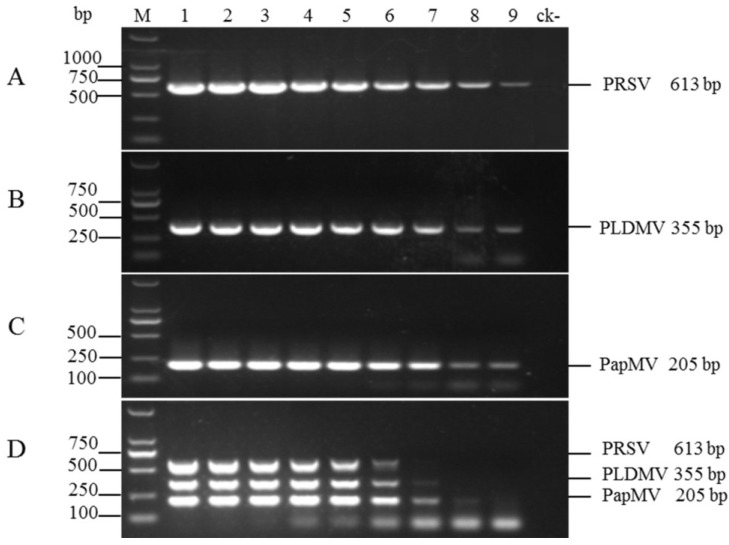
Comparison of the sensitivities of uniplex and multiplex RT-PCR assays for the detection of PRSV, PLDMV, and PapMV. Fragments 613 bp, 355 bp, and 205 bp of PRSV (**A**); PLDMV (**B**); and PapMV (**C**), respectively, were amplified from 10-fold serial dilutions of a mixture of plasmids containing inserts of target genes of three viruses. Lane 1–9: 10^9^–10^1^ copies; (**A**)–(**C**), uniplex RT-PCR; (**D**) multiplex RT-PCR. Lane ck-: health plants as a negative control. Lane M: DL2000 DNA Marker.

Sensitivities of uniplex RT-PCR and multiplex RT-PCR for the detection of three papaya viruses were determined by using 10-fold serial dilutions of plasmid pPRSV-613, pPLDMV-355, and pPapMV-205 (6.0 to 6.0×10^−8^ ng). The uniplex RT-PCR was able to detect template up to 1.44 × 10^1^ copies, 1.79 × 10^1^ copies, and 1.91 × 10^1^ copies for PRSV, PLDMV, and PapMV, respectively (lane 9 in Figure 4A–C), whereas the detection limits of the multiplex RT-PCR were 1.44 × 10^3^ copies, 1.79 × 10^3^ copies, and 1.91 × 10^2^ copies for PRSV, PLDMV, and PapMV, respectively (lane 7, lane 7 and lane 8 in Figure 4D). These results indicated that the multiplex RT-PCR was only 10 or 100-fold less sensitive than the uniplex RT-PCR.

### 3.4. Evaluation of Multiplex RT-PCR Using Field Samples

**Figure 5:**
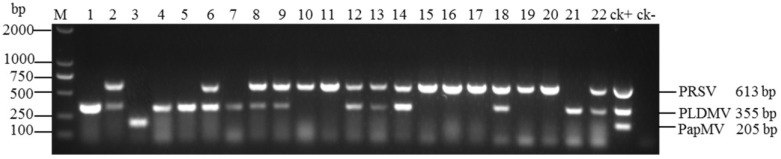
Agarose gel electrophoresis of 22 field samples collected from Hainan Island in plantations, kitchen gardens, and feral patches by multiplex RT-PCR. Various lanes show papaya plant infected with PRSV and PLDMV (Lanes 2, 6, 8, 9, 12, 13, 14, 18, and 22); PRSV (Lanes 10, 11, 15, 16, 17, 19, and 20); PLDMV (Lanes 1, 4, 5, 7, and 21); PapMV (Lane 3); Lane ck+: positive control, PRSV + PLDMV + PapMV. Lane ck-: health plants as a negative control. Lane M: DL2000 DNA Marker.

The multiplex RT-PCR was validated for its practical application by detecting PRSV, PLDMV, and PapMV using 341 field samples (Table 2 and Figure 5). PRSV, PLDMV, and PapMV were detected in 186/341 (54.5%), 93/341 (27.3%), and 3/341 (0.9%), respectively. Mixed infection of PRSV and PLDMV was detected in 59/341 (17.3%) of the samples. All of those field-collected samples were further confirmed using uniplex RT-PCR and the data showed that the results were in agreement with the multiplex RT-PCR (Table S1), indicating that the multiplex RT-PCR assay developed in this study is a rapid and validated method.

**Table 2:** Detection of Papaya ringspot virus (PRSV), Papaya leaf distortion mosaic virus (PLDMV), and Papaya mosaic virus (PapMV) in papaya plants from different geographical regions of Hainan Island.

Geographical Region	No. of Samples	PRSV Only	PLDMV Only	PapMV Only	PRSV and PLDMV (Mixed Infection)
Haikou	30	15(50%)	8(26.7%)	1(3.3%)	6(20%)
Sanya	28	17(60.7%)	8(28.6%)	0(0%)	3(10.7%)
Wenchang	16	10(62.5%)	3(18.8%)	0(0%)	3(18.8%)
Qionghai	13	7(53.8%)	2(15.4%)	0(0%)	4(30.8%)
Wanning	9	6(66.7%)	2(22.2%)	0(0%)	1(11.1%)
Wuzhishan	13	8(61.5%)	3(23.1%)	0(0%)	2(15.4%)
Dongfang	34	9(26.5%)	13(38.2%)	1(2.9%)	11(32.4%)
Danzhou	12	8(66.7%)	3(25%)	0(0%)	1(8.3%)
Lingao	15	11(73.3%)	4(26.7%)	0(0%)	0(0%)
Chengmai	13	6(46.2%)	4(30.8%)	0(0%)	3(23.1%)
Dingan	16	16(100%)	0(0%)	0(0%)	0(0%)
Tunchang	14	10(71.4%)	2(14.3%)	0(0%)	2(14.3%)
Changjiang	20	14(70%)	4(20%)	0(0%)	2(10%)
Baisha	12	9(75%)	1(8.3%)	0(0%)	2(16.7%)
Qiongzhong	18	8(44.4%)	8(44.4%)	0(0%)	2(11.1%)
Lingshui	19	11(57.9%)	7(36.8%)	0(0%)	1(5.3%)
Baoting	21	10(47.6%)	6(28.6%)	0(0%)	5(23.8%)
Ledong	38	11(28.9%)	15(39.5%)	1(2.6%)	11(28.9%)
Total	341	186(54.5%)	93(27.3%)	3(0.9%)	59(17.3%)

## 4. Discussion

A PCR-based method is routinely used to diagnose the presence of plant pathogens, particularly plant viruses. In this study, the uniplex and multiplex PCR and RT-PCR protocols were standardized for the simultaneous detection of PRSV, PLDMV, and PapMV, both as single and as mixed infections in papaya. Generally, primer pairs are the most important factors affecting the efficiency and specificity of a multiplex RT-PCR. Therefore, three primer pairs were designed according to the conserved region of the CP gene of PLDMV and PapMV. However, a different target *P3* gene was used for PRSV. Previous methods to detect PRSV targeted the *CP* gene, a conserved region of the nuclear inclusion protein (*NIb*) gene, or the 3’-UTR, which are relatively more highly conserved compared to other regions of the complete genomic sequence of PRSV [17,18,20,24]. However, these genes or fragments from different geographical PRSV isolates have been used to develop transgenic cultivars with resistance to PRSV in many countries, such as China, America, Brazil, Jamaica, Venezuela, Thailand, Australia, and some countries of East Africa [43,44,45]. Indeed, two transgenic papaya lines have been planted commercially in Hawaii, United States, since 1998, and in Guangdong province, China, in 2006 [43,45,46]. Therefore, the primers previous designed could not be used to distinguish between transgenic papaya with PRSV *CP* or *NIb* gene for the resistance against PRSV and PRSV-infected papaya. In this study, a highly conserved region (2774–4207 nt) within the *HC-Pro* and *P3* gene was chosen as a suitable target to design three primer pairs specific for PRSV through aligning 26 isolates of the complete genomic sequence of PRSV by software. Of the six primers screened in uniplex and multiplex RT-PCR, the primer combination PRSV613-F/R + PLDMV355-F/R + PapMV205-F/R was selected for further optimization in multiplex RT-PCR. The three amplicons (613 bp for PRSV, 355 bp for PLDMV, and 205 bp for PapMV) were easily differentiated on agarose gel electrophoresis since their size difference was 150 bp or more.

In this work, 2 × *Eco Taq* PCR SuperMix (+dye) (TransGen Biotech) including optimized PCR buffer, *Taq* DNA polymerase, dNTP, and loading dye was used to optimize the reaction systems and conditions. Currently, premixed master *Taq* DNA polymerase is supplied by most biotechnology companies worldwide, and successfully amplifies low-titer templates even in the presence of inhibitors. This is among the most popular applications for routine PCR because of several advantages: (i) minimized PCR optimization steps with advanced buffer systems; (ii) enhanced enzyme stability for increasing yield and specificity of PCR amplification; (iii) using convenient master mix formats can save time and reduce the risk of sample contamination during assay setup; (iv) no need to add gel loading dye can save time on agarose gel electrophoresis. Therefore, by the absence of a need to optimize the concentrations of *Taq* DNA polymerase, dNTP, and Mg^2+^ in multiplex RT-PCR, the time, labor, and reagent costs in the detection of these three viruses was reduced, especially for a large number of samples.

A frequent problem in multiplex RT-PCR is the unbalanced amplification of certain viruses due to the presence of multiple targets in one reaction and to primers with different compatibility to their targets, which may result in competition for enzymes and nucleotides in the reactions [23,47]. In this study, the simultaneous amplification of PRSV, PLDMV, and PapMV by using mixtures of equal amounts of primer pairs specific for those three viruses resulted in strong differences in band intensity on the agarose gel. Further, the strong amplification of the PLDMV band of 355 bp was produced in all multiplex RT-PCR assays (Figure 3, Lane 1–10). To obtain balanced amplification efficiency of all three viruses, plasmids containing the target genes of those three viruses were used for the optimization of primer concentrations, which ensured that similar band intensities resulted from almost equal amounts of templates. Thus, the optimum combinations of the primer concentrations were 0.4 μM for PRSV, 0.1 μM for PLDMV, and 0.2 μM for PapMV in the multiplex RT-PCR (Figure 3, Lane 7). In addition, the assay sensitivity of multiplex RT-PCR compared to uniplex RT-PCR was approximately 10-100-fold lower for detection of PRSV, PLDMV, and PapMV. Similar findings were reported in other studies [23,32,34,48], which owe to some factor affecting a multiplex RT-PCR efficiency, such as high oligonucleotide and primer concentrations.

Validation of this approach in 341 field samples collected from 18 counties covering Hainan Island identified infection by PRSV, PLDMV, and PapMV in 186/341 (54.5%), 93/341 (27.3%), and 3/341 (0.9%), respectively. Furthermore, 59/341 (17.3%) of the samples were co-infected with PRSV and PLDMV, which is the first time being reported in Hainan Island. These results have shown that PRSV has a high prevalence and is a major papaya pathogen on Hainan Island, China. Additionally, PLDMV was detected in 18 counties, representing nearly the entire island, but it was reported for the first time in Dongfang in 2012 [14], which suggested that PLDMV might be nearing outbreak. Furthermore, the mixed infection with PRSV and PLDMV has been detected by this multiplex RT-PCR. In contrast, PapMV infection remains infrequent. Thus, viral diseases in papaya become more and more complex and papaya production likely faces important new challenges concerning the transmission of PRSV and PLDMV in China.

In conclusion, this is the first report of multiplex RT-PCR for the diagnosis of PRSV, PLDMV, and PapMV. A reliable, specific, sensitive, and cost-effective multiplex RT-PCR technique was developed for detection and differentiation of these three viral infections in samples of papaya. This technique is useful for routine molecular diagnosis and epidemiological studies of papaya. The results of field application of this multiplex RT-PCR method revealed that the major limiting factor for papaya production in Hainan Island comes from separate or combined infections of PRSV and PLDMV.

## Supplementary Files

Supplementary File 1
