# Supplementary material for: Development and Validation of a Multiplex Reverse Transcription PCR Assay for Simultaneous Detection of Three Papaya Viruses
Source: Viruses. 2014 Oct 21;6(10):3893–906. doi: 10.3390/v6103893 (PMC4213569; doi:10.3390/v6103893)
Supplement: Supplementary File 1 [file viruses-06-03893-s001.pdf]

Supplementary Materials

Development and Validation of a Multiplex Reverse Transcription PCR Assay for Simultaneous Detection of Three Papaya Viruses

Decai Tuo, Wentao Shen, Yong Yang, Pu Yan, Xiaoying Li and Peng Zhou

Table S1. Comparison of the detection efficiency for PRSV, PLDMV, and PapMV in 341 field samples by uniplex RT-PCR and multiplex RT-PCR.

Virus	No. of field samples	uniplex RT-PCR		multiplex RT-PCR		Co-efficiency rate %
		Positive no.	Positive rate %	Positive no.	Positive rate %	
PRSV	341	245	71.8	245	71.8	100
PLDMV	341	152	44.6	152	44.6	100
PapMV	341	3	0.9	3	0.9	100
